# X Inactivation Lessons from Differentiating Mouse Embryonic Stem Cells

**DOI:** 10.1007/s12015-015-9597-5

**Published:** 2015-07-22

**Authors:** Greta Pintacuda, Andrea Cerase

**Affiliations:** Department of Biochemistry, University of Oxford, Oxford, OX1 3QU UK; EMBL Mouse Biology Unit, Monterotondo, 00015 RM Italy

**Keywords:** Stem cell biology, Epigenetics, Cell differentiation, X inactivation

## Abstract

X chromosome inactivation (XCI) is the dosage compensation mechanism that evolved in female mammals to correct the genetic imbalance of X-linked genes between sexes. X chromosome inactivation occurs in early development when one of the two X chromosomes of females is nearly-completely silenced. Differentiating Embryonic Stem cells (ESC) are regarded as a useful tool to study XCI, since they recapitulate many events occurring during early development. In this review we aim to summarise the advances in the field and to discuss the close connection between cell differentiation and X chromosome inactivation, with a particular focus on mouse ESCs.

**Electronic supplementary material:**

The online version of this article (doi:10.1007/s12015-015-9597-5) contains supplementary material, which is available to authorized users.

X Chromosome inactivation is the mechanism by which -therian mammals compensate for the genetic diversity between males (XY) and females (XX) in relation to the genes located on the X chromosome. In mouse development, two waves of X chromosome inactivation have been described. A first wave occurs from the 2- to 4-cell embryonic stage onward, and it is known as imprinted X inactivation (iXCI) [1]. Imprinted X inactivation always results in the paternal X being silenced, and it is regarded as the ancestral form of XCI. Indeed, it is the only form of XCI in marsupials, in which it appears to be incomplete and prone to reactivation [2]. In contrast with marsupials, placental mammals stably maintain the silencing of the paternal X only in those cells that will form the extra-embryonic tissues. At the blastocyst stage, the silencing is in fact reverted and the active state of both Xs is re-established in the Inner Cell Mass (ICM). After implantation, the ICM forms the epiblast, which originates the embryo proper, and the primitive endoderm [3]. During epiblast formation, one of the two X chromosomes is randomly selected to be inactivated, in a process known as random X inactivation (rXCI) [1, 4]. Both random and imprinted XCI depend upon a long non-coding RNA (lncRNA) called Xist (Inactive X specific transcript), which acts as the master regulator of the process [5]. Differentiating female ESCs are an excellent model for studying rXCI as they closely recapitulate the sequence of events observed in the developing embryo [6, 7]. In this review, we focus on what we have learned about rXCI from ESC models in the context of cell differentiation, at the chromatin, chromosomal and nuclear level. As significant differences in XCI [8, 9] and stem cell biology (see also Box 1) [10–12], have been described across different mammalian species, in this review we refer specifically to mouse XCI and to the interplay between the two major lncRNAs regulating XCI, Xist and Tsix. Since other non-coding RNAs have been shown to regulate XCI, readers are encouraged to consider the following reviews and articles on the topic [13–15].

**Table:** 

**Box 1**
**Totipotency**
It is defined as the capability of a given cell to differentiate into all cell types in the body. Early stages of dividing zygotes are an example of totipotent cells.
**Pluripotency**
Pluripotency is defined as the potential of a cell to generate different cell types. The greater the number of different cell types, the greater the pluripotent capacity of the cell. ESCs are pluripotent because they cannot generate extra embryonic tissues.
**Pluripotency factors**
Pluripotency factors are a set of transcription factors that regulate the pluripotent status of the cell by transcriptional (and co-transcriptional) regulation of pluripotency-associated genes.
**Mouse vs. human embryonic stem cell X chromosome inactivation**
There are several differences between human ESC (hESC) and mouse ESC (mESC) in the context of XCI. Indeed, while mouse ESC have two active X chromosomes, conventional human ESCs have an active and an inactive chromosome. However, a näive state of hESC was also described where either two active X chromosomes present, mix of an active and inactive ones can be observed. Very importantly, cell culture conditions can be adjusted to enrich for two active X chromosomes.
**Polycomb group proteins (PcGs)**
Originally discovered in Drosophila as essential regulators of Hox genes and body development, they play an essential role in cell differentiation.
**Self-renewal**
It is defined as the capability of a given cell to go through a number of divisions while maintaining its identity. Self-renewal is a hallmark of cell types like ESCs and cancer cells.
**ESC culturing**
In standard conditions, embryonic stem cells (ESCs) are cultured in high-serum, LIF-containing medium. Additionally, they can be grown on fibroblast feeder cells which help maintain ESC pluripotency. Alternatively, ESC medium can be replaced by 2i medium which contains LIF and 2 inhibitors of differentiation, one blocking the mitogen-activated protein kinase signalling (Mek) and the other inhibiting glycogen synthase kinase-3 pathway (Gsk3).
**X chromosome inactivation (XCI)**
XCI is divided into two stages: The **Establishment and the Maintenance phase**. During the establishment phase, Xist RNA is transcriptionally up-regulated on one of the two X chromosomes, it spreads *in cis* (on the same chromosome it is transcribed from) and induces gene silencing by recruitment of chromatin and DNA modifiers. Once the silent state has been established it is maintained through subsequent cell divisions (maintenance phase) by the multiple layers of epigenetic modifications present on the inactive X (i.e. repressive histone modifications, histone exchange, DNA methylation, etc.). Importantly, maintenance of X chromosome inactivation appears to be largely Xist independent.
**X inactivation models**
Different model systems have been used to study XCI and its role in mammalian development. Differentiating **female (XX) ESCs** represents one of the best model systems for studying XCI. In fact, this system recapitulates quite closely the early stages of XCI described in the embryo. Female ESCs can be differentiated using retinoic acid, or via LIF (an anti-differentiation factor) withdrawal. Retinoic acid (RA) treatment induces transcription of specific target genes triggering the cell to differentiate. Culturing cells in the absence of LIF (LIF removal) leads to **Embryoid Body (EB)** formation. EBs are three-dimensional multicellular aggregates that may have a non-homogeneous cell composition. **Transgenic ESCs (XX or XY)** bearing an inducible *Xist*-transgene (*Xist*-Tg) are another widely-used model in XCI research. Finally, **Mouse Embryonic Fibroblasts (MEFs)** are regarded as a good model for the late steps of XCI as they are terminally differentiated cells in which X inactivation has already occurred (maintenance phase).
**Nuclear matrix**
The **nuclear matrix** (or **nuclear scaffold**) is a stable, mash-like proteinaceous structure providing the framework for chromatin organization.
**Cell Reprogramming**
Fully differentiated cells can be reprogrammed to an ESC-like state by transfecting them with a cocktail of transcription factors (Yamanaka’s protocol), by fusion of differentiated cells and ESCs, or by nuclear transfer. Noticeably, reprogrammed female ESCs exist with either 2 active X chromosomes or one active and one inactive X chromosome.

ESCs are characterised by their ability to perpetuate the pluripotent state and to self-renew. This is achieved through multiple signalling pathways, transcription factor activities, and the inhibition of differentiation. In undifferentiated ESCs, expression of the so-called “pioneer pluripotency factors” Oct-4, Sox2, and Nanog [16] (Fig. 1a) is critical. These genes regulate the pluripotent state by acting on thousands of pluripotency-associated target genes [19, 20]. The Jak-Stat3 signalling pathway is also essential to keep ESCs in an undifferentiated state. In particular, the activation of the Jak-Stat3 pathway by the Leukemia inhibiting factor (LIF) is necessary and sufficient both to maintain the pluripotent state and to perpetuate self-renewal [20]. Moreover, pluripotency and self-renewal can also be maintained by suppressing the signalling cascades needed for cells to differentiate. In this regard, the Smith's group has shown that cells can be kept in an undifferentiated state, despite external stimuli, by inhibiting the Mek and Gsk3 signalling pathways (2i conditions) [21, 22].

**Fig. 1:**
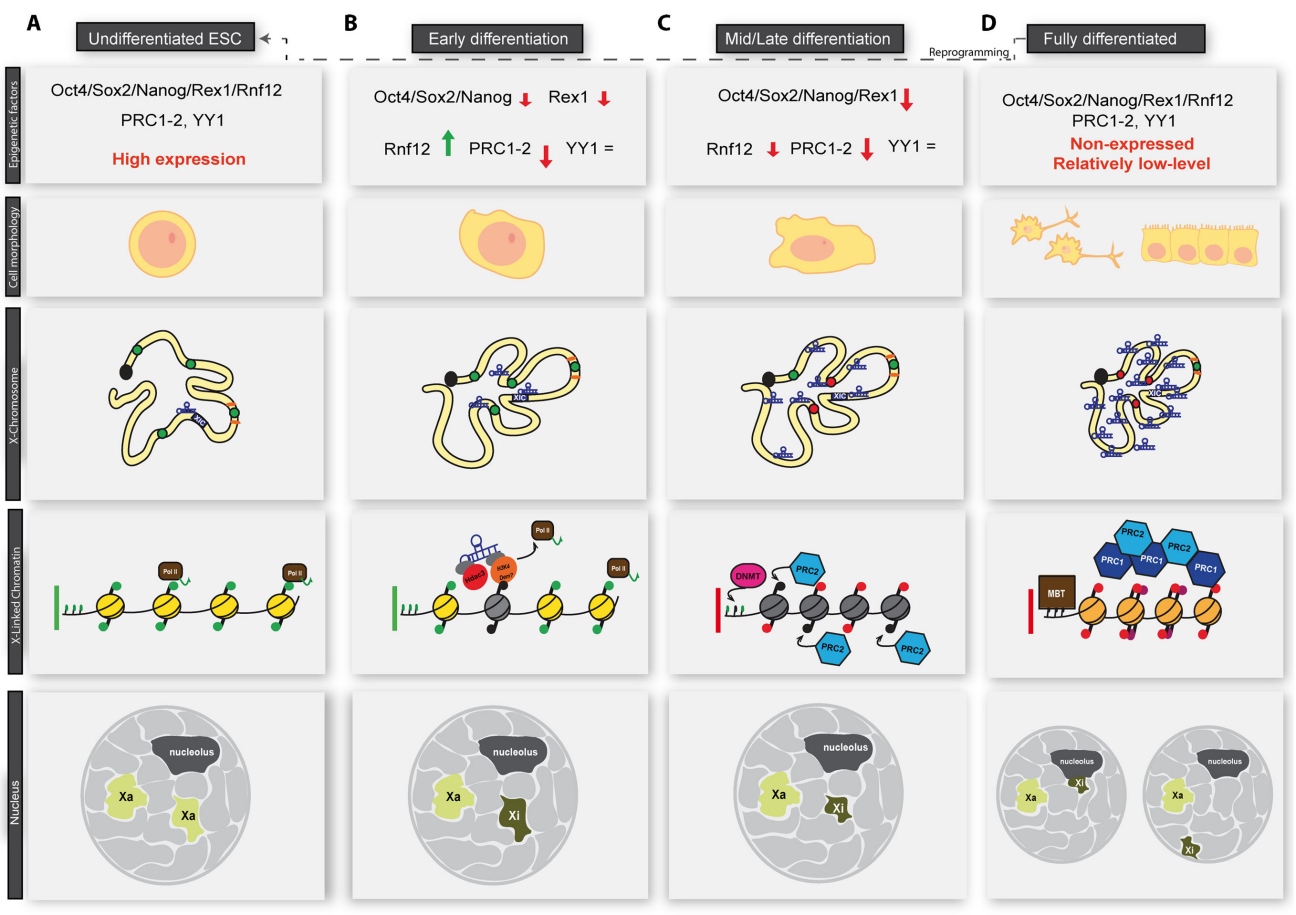

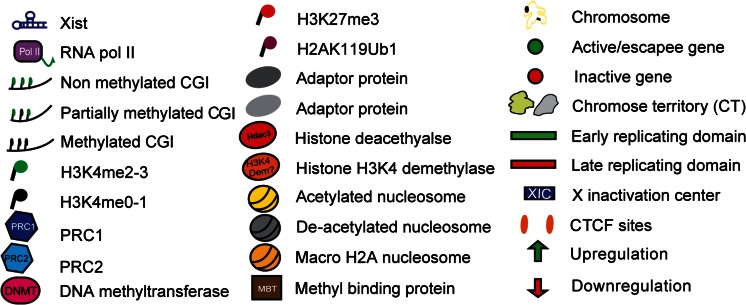
**a**) In undifferentiated ESCs, chromatin is decompacted and cells are in a fully pluripotent state. Pluripotency factors, master epigenetic regulators (i.e. Polycomb proteins, PRC1/2) are highly expressed and most of the genome is early replicating. **b**) Early in differentiation, pluripotency factors are downregulated, allowing the monoallelic upregulation of Xist on the future inactive X (Xi). PRC1/2 complexes are also dowregulated [16], YY1 levels remain constant during differentiation [17]. Xist spreads in the 3D neighbourhood and recruits chromatin modifiers like histone deacetylases and/or H3K4me2-3 demethylases to the future inactive X. This recruitment can be either direct or mediated by an adaptor protein. As a consequence of Xist activity, RNA pol II is displaced from actively transcribing promoters. Genes to be silenced start to be relocated inside the Xist-repressive compartment. **c**) Removal of RNA Pol II from chromatin allows the recruitment of Polycomb proteins (PcGs) and DNA methyltransferases (DNMTs). In particular, the future inactive X becomes enriched for the PRC2 mark H3K27me3 and begins to get compacted as a consequence of gene silencing. Gene relocation is nearly complete at this stage, with only few escapee genes not internalised. CTCFs may serve as a barrier to protect escapee genes. The future inactive X also becomes late replicating. **d**) PRC2 mark (H3K27me2-3) is in turn recognised by PRC1 and this silencing loop is reinforced by the addition of H2A119ub1* and histone H2A is replaced by the silencing-associated histone variant, macroH2A. Xist spreading is complete at this stage. Chromatin compaction has reached its maximal level and the inactive X translocates to the proximity of the nuclear lamina or the nucleolus. In fully differentiated cells, pluripotency factors are very low. PRC1/2 levels are also usually low. Cell reprogramming can revert the differentiated state to an ESC-like state (iPSC), which is compatible with de novo XCI establishment. *Note: PRC1 can be recruited to the inactive X independently of H3K27me3 mark and it starts to accumulate at low levels on the inactivating X at a similar time [18]

Mouse female ESCs have two active X chromosomes, as the ICM cells from which they derive [23], and their chromatin is mostly de-compacted (Fig. 1a) [24]. Transcriptional upregulation of Xist represents the molecular switch that triggers XCI. This event is regulated by pluripotency factors [25] and other non-coding RNAs (ncRNA) located in the X-inactivation center (XIC) [26]. Tsix, for instance, is a long non-coding RNA (lncRNA) antisense to Xist, and the main antagonist to Xist transcriptional activation [27, 28]. Therefore, it is the relative ratio of Xist/Tsix expression which appears to control the initiation of XCI.

When female ESCs differentiate (Fig. 1b), there is a stochastic fluctuation of the level of pluripotency factors and master epigenetic regulators such as Polycomb repressive complexes 1–2 (PRC1-2) [16, 29]. In particular, Oct4 and Nanog, acting as Xist repressors, are downregulated, resulting in an increase of Xist transcription [30, 31], although the exact molecular mechanism is not entirely understood [32, 33]. In this time window, Rnf12, an X-linked ubiquitin ligase, is upregulated and targets for proteosomal degradation Rex1, an important Tsix activator and Xist repressor [34]. Rex1 degradation allows the binding on the Xist promoter of the Xist activator YY1, with which competes for binding sites, resulting in increased Xist expression [17]. The combination of these differentiation-induced changes, shifts the balanced level of expression of Xist and Tsix towards a strong mono-allelic upregulation of Xist, initiating, de facto, XCI [34]. It is important to highlight here that the exact role of Rnf12 in rXCI remains controversial. Indeed, although its up-regulation in male ESCs leads to abnormal XCI, female Rnf12+/− mice and ESCs are still able to normally inactivate the X chromosome, suggesting that additional factors acting downstream and possibly independently of Rnf12 might be involved in the process [35]. Moreover, in vitro and in vivo experiments carried out in mouse and other species show that monoallelic Xist upregulation may not be happening in all differentiating cells [8, 36]. Indeed, cells silencing more than one X chromosome would disappear due to counter-selection [8, 36].

Exploiting a transcriptionally favourable window of opportunity, Xist starts to spread *in cis* using local spatial proximity (Fig. 1b) [37, 38]. 3D proximity is, therefore, the major determinant of Xist initial spreading over any genomic feature or chromatin signature [37, 38]. How Xist is able to spread only *in cis* is still unknown and remains an object of debate. However, it is known that HnrnpU/Saf-A, a nuclear scaffold protein, is necessary for Xist localisation on the inactive X (Xi) [39]. A recent paper [40] confirmed the direct interaction between Xist and Saf-A and suggested a possible role a post-translationally modified form of Saf-a [41, 42]. Alternatively, Xist could be post-transcriptionally modified (i.e. by RNA methylation) and the regulation of this process might be important for Xist *in cis*-spreading and binding to silencing partners [43]. Noticeably, the mechanism through which Xist induces gene silencing is also still unclear. However, we know that among the earliest events triggered by Xist up-regulation are global histone deacetylation and the removal of H3K4me2-3 marks [44, 45]. As these two events are the earliest detected, it is possible that Xist directly or genetically interacts with a histone deacetylase complex and/or a lysine demethylase complex but not with the PRC2 complex, which is recruited after these events [44, 46, 47]. Another early hallmark of Xist silencing is the exclusion of RNA Polymerase II from the presumptive inactive X chromosome territory [44]. This can be a direct consequence of histone deacetylation and/or H3K4me2-3 demethylation [48]. In fact, the transcription-permissive H3K4me3 mark [49, 50] is recognised by TAF3 and plays a role in the recruitment of the transcription machinery at engaged promoters [51]. PRC2 is also recruited to the inactive X by the PRC2 co-factor Jarid2 [52] and at this stage of differentiation PRC2 recruitment is completely Xist-dependent [53].

Xist expression and localisation during differentiation to determines which X chromosome will be inactivated. Nevertheless, at this state XCI is fully reversible [53].

As cells differentiate, the X chromosome, like like any other chromosome, rearranges itself according to the specific lineage commitment of each cell type [54, 55] (Fig. 1c). Nanog downregulation, in particular, seems to be crucial for priming cells towards differentiation [56], although Nanog is itself dispensable for somatic stem cell pluripotency, as are the Polycomb Group Repressive Complexes 1–2 (PcGs/PRC1-2) [57, 58]. During this phase, X-linked genes to be silenced are actively relocated into the Xist-silent compartment, with the exception of few escapee genes looping out of it [44]. CTCF binding may serve as a barrier to prevent escapee genes being internalised into the inactive compartment [59]. Around day 2–3 of differentiation, Oct-4 and Sox2 are also downregulated to different extents, depending on the cell differentiation fate [16]. Namely, cells with higher Sox2 will become neuronal ectoderm and cells with higher Oct-4 mesoderm [55, 60]. The future inactive X becomes late replicating [61, 62], while promoter DNA methylation starts to be established, with different dynamics on a gene-to-gene basis, depending on Smchd1 activity [63]. Moreover, higher chromatin compaction of the inactivating X chromosome starts to become visible [40]. As differentiation proceeds, X inactivation becomes fully irreversible, mostly due to the differentiation/committed state of the cells [53].

In fully differentiated cells (Fig. 1d), Xist broadly localises onto the inactive X chromosome and is mostly enriched on gene-rich, LINE-poor regions [37, 38]. Gene silencing is stably maintained by the redundant action of multiple layers of epigenetic modifications, such as DNA methylation, Methyl-CpG-Binding proteins (MBDs), late replication timing, macro H2A incorporation, PRC1/2 activity [18] and repressive histone modifications [6, 64, 65]. PRC1/2 accumulation is now stably maintained on the inactive X as a consequence of gene silencing [48] and a self-reinforcing positive feedback loop [66]. The inactive X has also reached the maximum level of chromatin compaction [40], and it is now positioned in the proximity of the nucleolus or the nuclear envelope [13, 67]. Its position in the nucleus seems to have a role in maintaining the silent state [67].

 The new state is, therefore, locked throughout the life of the individual. Only in the primordial germ cells (PGCs) is reverted and two active X chromosomes co-exist in the same nucleus [68]. Maintenance of the silent state is now largely Xist-independent [69, 70].

A significant open question in the field is understanding why Xist expression is not capable of setting up de novo gene-silencing in fully differentiated cells [53]. The reason for Xist lack of competency in this context is not completely clear but it is tempting to speculate that might be due to either the absence or insufficient expression of accessory proteins required to establish gene silencing [71]. In this regard, cell reprogramming experiments (Fig. 1) can revert a fully differentiated cells into induced Pluripotent Stem cells (iPSc), in which Xist-silencing competency is restored [19]. The transition from fully differentiated cells to iPSc is interesting in the light of XCI reactivation. In fact, this process seems to pass through a series of intermediate states that might be useful for uncovering new links between Xist silencing and differentiation [72] and finding new potential XCI players. For an overview of XCI reversal during cell-reprogramming, the readers are referred to the following reviews [12, 73, 74].

In conclusion, we believe that better knowledge of how Xist works will come from discovering and dissecting the molecular machinery interacting with Xist during the favourable developmental time window in which silencing is established [43, 47]. Therefore, studying the role of the proteins that directly or genetically interact with Xist is crucial for a better understanding of XCI. We predict that these proteins may also have a key role in cell differentiation.

## Electronic supplementary material

ESM 1 (GIF 931 kb)

High Resolution Image (TIFF 49.8 mb)

## Glossary

2i medium: 2 inhibitor embryonic stem cell (ESC) medium
CT: Chromosomal territory
CTCF: CCCTC-binding factor
ESC: Embryonic stem cells
hESC: Human embryonic stem cells
H3K4me2-3: Histone 3 lysine 4 di-trimethylation
H3K27me3: Histone 3 lysine 27 trimethylation
ICM: Inner cell mass
iPSC: Induced pluripotent cells
iXCI: Imprinted X chromosome inactivation
lncRNA: Long non-coding RNA
LIF: Leukemia inhibitory factor
mESC: Mouse embryonic stem cells
MDB: Methyl-CpG-binding protein
ncRNA: Non-coding RNA
PcG: Polycomb group proteins
PGC: Primordial germ cells
Pol II: RNA polymerase II
PRC1-2: Polycomb repressive complex 1 & 2
rXCI: Random X chromosome inactivation
SAF-A/hnRNP: U scaffold attachment factor A/heterogeneous ribonucleoprotein U
Tsix: Antisense transcript to Xist
Xa: Active X chromosome
Xi: Inactive X chromosome
XIC: X-inactivation center (a ~ 350 kb region around Xist)
Xist: Inactive X specific transcript
