# Supplementary figures and images for: X Inactivation Lessons from Differentiating Mouse Embryonic Stem Cells
Source: Stem Cell Rev. 2015 Jul 22;11(5):699–705. doi: 10.1007/s12015-015-9597-5 (PMC4561061; doi:10.1007/s12015-015-9597-5)

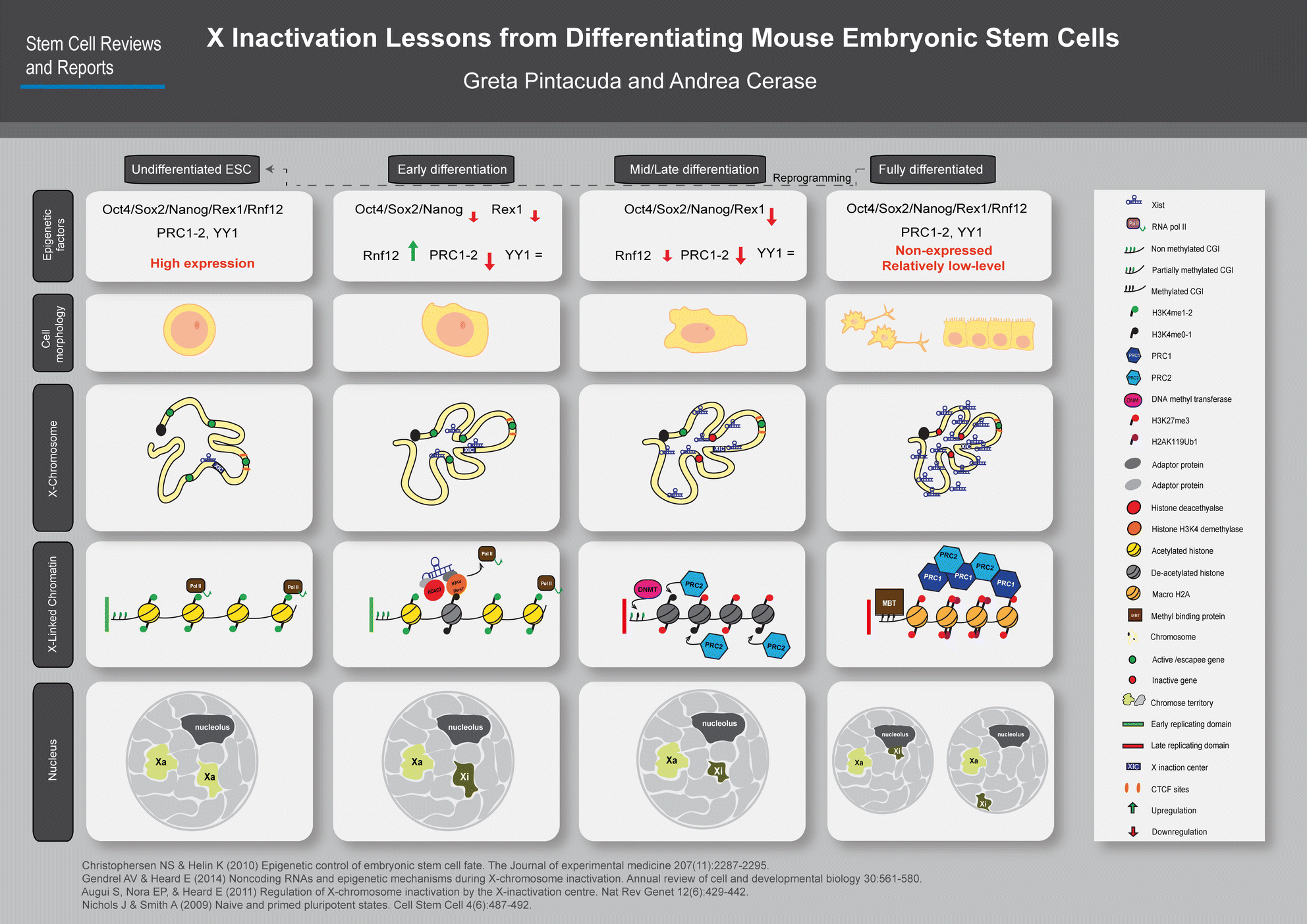

Supplement: Supplementary file 1 — (GIF 931 kb) [file 12015_2015_9597_Fig2_ESM.gif]

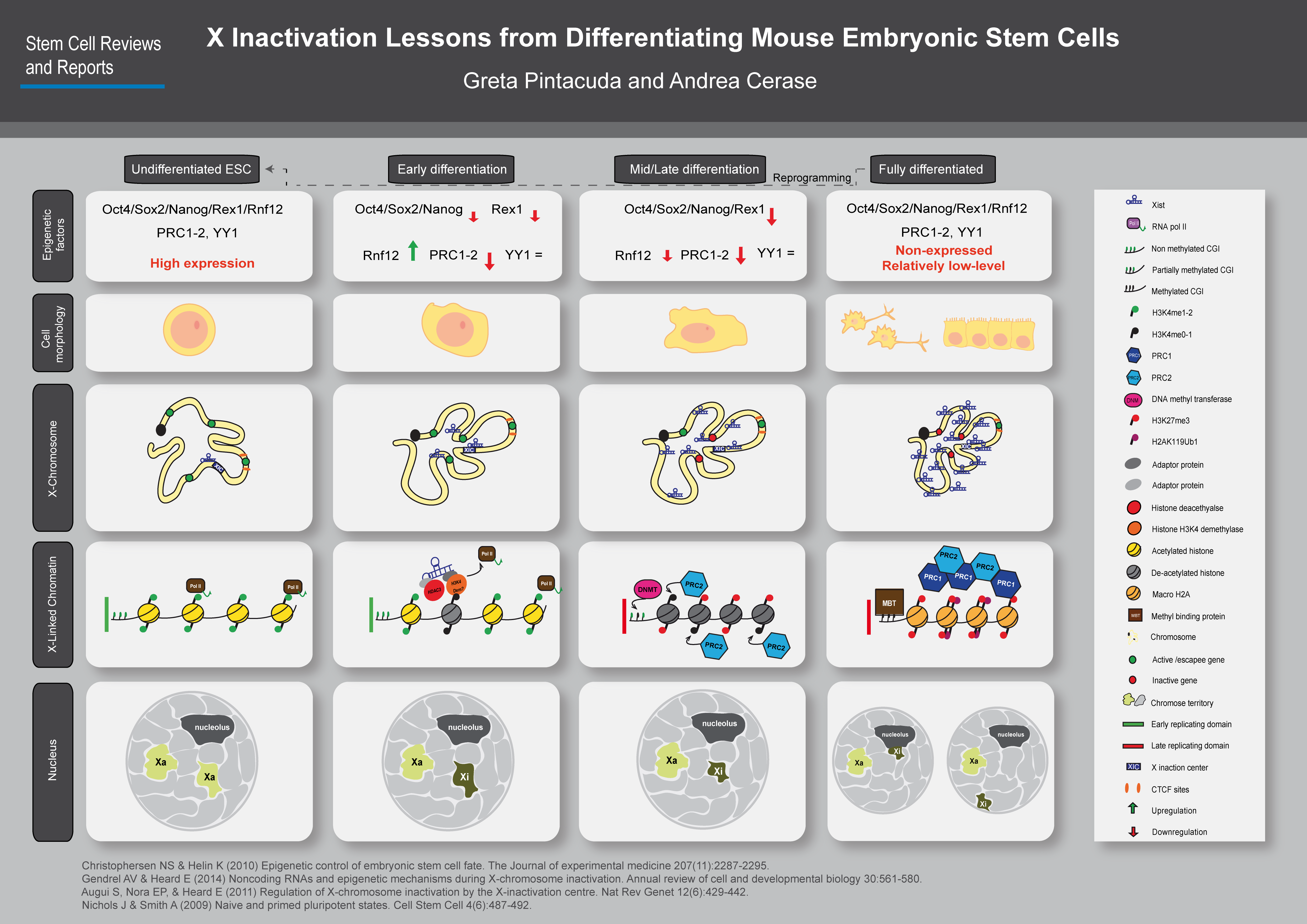

Supplement: Supplementary file 2 — High Resolution Image (TIFF 49.8 mb) [file 12015_2015_9597_MOESM1_ESM.tif]
